# CCN Proteins as Matricellular Regulators of Bone in Aging and Disease

**DOI:** 10.1007/s11914-025-00915-4

**Published:** 2025-05-23

**Authors:** Parveez Ahamed Abdul-Azees, Rahul Rajesh, Travis J. Block, David D. Dean, Chih-Ko Yeh, Maegan Capitano, Melissa Kacena, Xiao-Dong Chen, Miloš Marinković

**Affiliations:** 1 Department of Comprehensive Dentistry, University of Texas Health Science Center at San Antonio, San Antonio, TX 78229 - 3900 USA; 2 Arkansas College of Osteopathic Medicine, Fort Smith, AR 72916 USA; 3 Research Service, South Texas Veterans Health Care System, San Antonio, TX 78229 USA; 4 Geriatric Research, Education and Clinical Center, South Texas Veterans Health Care System, San Antonio, TX 78229 USA; 5 Department of Microbiology and Immunology, Indiana University School of Medicine, Indianapolis, IN 46202 USA; 6 Department of Orthopaedic Surgery, Indiana University School of Medicine, Indianapolis, IN 46202 USA; 7 Department of Anatomy, Cell Biology and Physiology, Indiana University School of Medicine, Indianapolis, IN 46202 USA; 8 Indiana Center for Musculoskeletal Health, Indiana University School of Medicine, Indianapolis, IN 46202 USA; 9 Department of Biomedical Engineering, University of Texas at San Antonio, San Antonio, TX 78249 USA; 10 Research Service, Richard L. Roudebush VA Medical Center, Indianapolis, IN 46202 USA

**Keywords:** Aging bone marrow, Matricellular signaling, Extracellular matrix, Mesenchymal stem cells, Osteoblasts, Osteoclasts

## Abstract

**Purpose of Review:**

This review explores the role of cell communication network (CCN) proteins in regulating skeletal physiology, aging, and disease, particularly within the context of balanced bone remodeling.

**Recent Findings:**

Recent conceptualization of paracrine and endocrine networks in bone marrow as a form of osteoimmunological crosstalk suggests a significant role for matricellular signaling in regulating bone homeostasis. As multifunctional adapters of cell–matrix interactions, CCNs are emerging as a focal point for parathyroid hormone (PTH) signaling and regulation of the RANKL/RANK/OPG axis in skeletal aging. Altered bone marrow CCN expression creates a permissive environment for accelerated postmenopausal bone loss and may contribute to the pathogenesis of osteoporosis and other diseases related to skeletal aging.

**Summary:**

CCNs modulate fundamental signaling mechanisms in bone development, homeostasis and repair. During aging, dysregulation of CCNs may negatively affect skeletal health and contribute to disease progression. As a result, CCNs may constitute promising therapeutic targets for improving and maintaining aging bone health.

## Introduction

To support the multitude of biomechanical, metabolic and hematopoietic functions of the skeletal system, complex networks of regulatory feedback maintain bone homeostasis throughout life. During aging, deterioration of mineral density and microarchitecture compromises bone structure, while replacement of mineralized matrix by infiltrating adipose tissue degrades both skeletal integrity and hematopoietic function [1, 2]. While estrogen deficiency is a major driver of bone degeneration, skeletal aging proceeds through distinct mechanisms and its impact on the structure and function of bone significantly exacerbates the effects of sex hormone-deficiency during menopause and andropause [3]. For example, dysregulation of lineage commitment by bone marrow mesenchymal stem cells (MSCs) in geriatric bone not only impairs their function as an osteoblastic reservoir but may support increased osteoclast activity associated with both aging and skeletal degenerative pathologies such as osteoporosis [2, 4]. Postmenopausal estrogen decline dysregulates the RANK/RANKL/OPG axis and related parathyroid hormone (PTH) signaling, while mechanisms of aging are more closely associated with senescence of skeletal cells and osteoimmunological inflammation [5–7]. Finally, recent research suggests that aging-associated changes in the bone extracellular niche also contribute to the precipitous decline in bone mass and microarchitecture caused by decreases in sex hormone levels [8–11].

Proteins belonging to the six-member cell communication network (CCN) family have attracted interest due to their significant roles in musculoskeletal development and physiology. They are involved in the regulation of osteogenesis, chondrogenesis, and osteoclastogenesis, as well as mechanosensory signaling and fracture healing [12, 13]. Additionally, CCNs are implicated in inflammatory pathologies like osteoarthritis and rheumatoid arthritis, as well as in cancers affecting the musculoskeletal system and bone metastasis [14, 15]. Their dual roles in homeostatic bone regulation and degenerative processes associated with aging and disease make them attractive therapeutic targets [16, 17]. As matricellular signaling proteins, their cytosolic, as well as extracellular, localization implicates CCNs within a broad range functions related to transduction and integration of cellular signaling, while their modular organization suggests an intriguing possibility for simultaneous association of their distinct domains with multiple ligands, potentially giving rise to diverse functions despite a high degree of sequence homology within the CCN family [18]. Accordingly, results from phenotypic and cell-based studies of CCN signaling in bone have largely shown them to act as pleiotropic regulators of bone development and metabolism, which coordinate the activities of multiple cell types to maintain bone homeostasis [19].

This review outlines the contributions of CCN proteins in bone homeostasis and summarizes insights into their role in the dysregulation of PTH and RANK/RANKL/OPG signaling in aging and disease. Emerging evidence from our studies and others suggests that targeting CCNs within cells or the bone microenvironment may represent a promising therapeutic strategy for skeletal aging and degenerative disease [20–24]. Finally, we summarize obstacles and opportunities for studying CCN signaling in bone and promising therapeutic directions.

## The CCN Protein Family

**Fig. 1:**
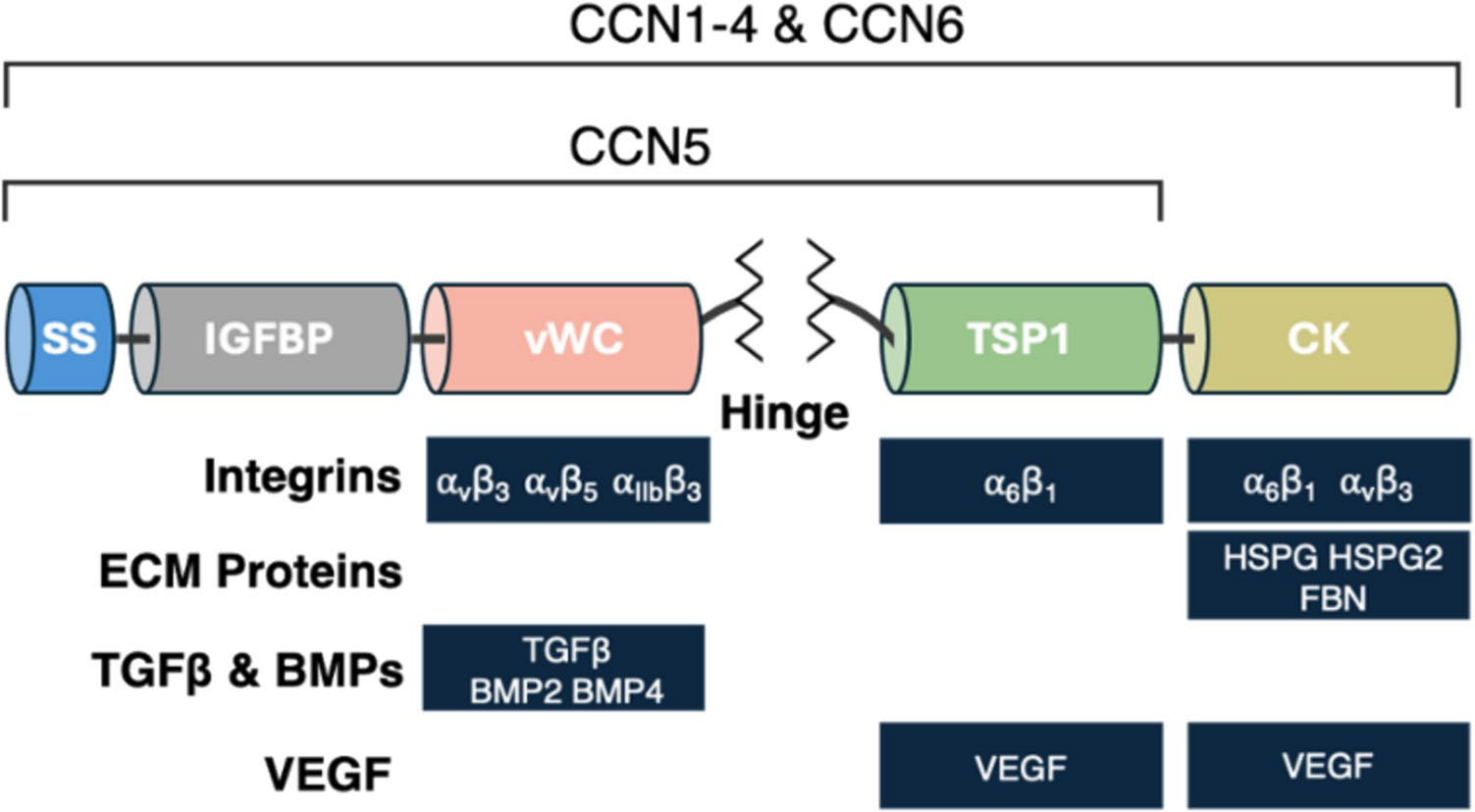
Generalized structural organization and binding domains of CCN proteins**.** With the exception of CCN5, all CCN family members are characterized by a highly-conserved structure, with each domain hosting a distinct set of interactions with various extracellular receptors, matrix proteins, or secreted growth factors. SS = secretory signal peptide; IGFBP = insulin-like growth factor binding domain; vWC = von Willebrand factor type C domain; TSP1 = a thrombospondin type 1 repeat domain; CK = carboxy-terminal cystine knot. The central hinge region varies considerably in length and organization between individual CCNs and determines vulnerability to proteolytic cleavage [27]

The CCN family consists of six (30–40 kDa) matricellular proteins, enriched in conserved cysteine residues. Each member contains four structural domains which interact with proteins in the cellular microenvironment, including the extracellular matrix (ECM), cell surface receptors, growth factors and cytokines (Fig. 1). While previously designated as Cyr61 (CCN1), CTGF (CCN2), NOV (CCN3), and WISP1 - 3 (CCN4 - 6), the unified “cell communication network” nomenclature was adopted to highlight their conserved sequence homology and role as non-structural matrix constituents [25]. Depending on their nuclear, cytosolic or extracellular matrix localization, CCN proteins direct cellular responses to growth factors, and have been shown to effect divergent outcomes (e.g., survival versus apoptosis), depending on the broader signaling context [26–31]. Despite their noted structural similarities, individual CCN proteins exhibit distinct, context-specific roles in regulating tissue homeostasis. For example, in human primary neonatal fibroblasts, CCN1 was shown to shift the role of TNF-α signaling from supporting osteoblast survival (via NF-κB) to promoting apoptosis via the JNK pathway by interacting with integrin α6β1 and syndecan- 4 at the cell surface [32]. Indeed, the unique role of CCNs to direct context-specific cellular responses to various growth factors is an intriguing aspect of their function, whose prospective therapeutic implications remain unrealized.

All CCN proteins (except CCN5) consist of a N-terminal secretory signal peptide (SS), followed by four distinct domains: an insulin-like growth factor binding domain (IGFBP), a von Willebrand factor type C domain (vWC), a thrombospondin type 1 repeat domain (TSP1), and a carboxy-terminal cystine knot (CK) [33]. The unstructured hinge region between the vWC and TSP1 domains accounts for a major source of variation between CCN1 - 6 [18]. Variations in length, organization, and flexibility of this unstructured region may affect the binding ability of adjacent domains, as well as their susceptibility to proteolytic cleavage. Interestingly, the notable absence of the CK domain in CCN5 (WISP2) may underlie unique signaling functions within the CCN family [34].

Although the expression of all six CCN proteins has been identified in the skeletal system, the specific functions of CCN1, CCN2, and CCN3 are currently most extensively described [19]. Overall, CCN1 and CCN2 are strongly associated with the regulation of bone remodeling, while CCN3 may act antagonistically and, in some instances, counterbalance the function of other CCNs. For example, CCN3 has been shown to impair osteogenic differentiation by inhibiting BMP-mediated Smad and MAPK signaling and suppress the Wnt pathway in certain contexts [35–37]. Beyond their emergent roles in supporting osteoblastic differentiation, CCN4 and CCN5 may be distinguished by expression in osteocytes [38]. In mature bone of CCN5 knockout (KO) C57BL/6J mice (CCN5.^Δex2−5::LacZ^), LacZ staining of cortical bone indicated concentrated CCN5 expression in the periosteum and has been suggested to be involved in mediating the skeletal stress response to mechanical loading [39]. Finally, while recent clinical case reports and cohort studies involving whole genome sequencing have linked CCN6 mutations to progressive pseudorheumatoid dysplasia (PPD), a rare genetic disorder that affects development of both bone and cartilage, its role in bone development and disease has not been explored as extensively as that in cartilage [40–44].

## Bone Phenotype in Genetically Modified CCN Mouse Models

The essential role played by CCNs in skeletal development and homeostasis is suggested by the conservation of genomic loci, protein structures, and amino acid sequences of the gene family across all vertebrate species [45]. Table 1 summarizes bone phenotypes observed in genetically engineered mouse models, which manipulate CCN expression, either at the germline or through tissue-specific transgenic constructs.

**Table 1:** Bone phenotypes of CNN-modified mice

Approach	Gene Targeting	Skeletal phenotype	Reference
CCN1			
KO Constitutive	CCN1^Δex1:LacZ−PGK−Neo^	Embryonic lethality	Mo et al. 2002 [46]
KO Conditional	CCN1^fl/fl^;Tg(Ocn-Cre)	Decreased trabecular and cortical bone mass; reduced bone anabolic response to PTH	Zhao et al. 2018 [47]; Zhao et al. 2020 [48]
CCN2			
KO Constitutive	CCN2^Δex1::PGK−NeopA^	Defective skeletal development, matrix synthesis, mineralization, ossification; impaired osteoblast maturation and function	Ivkovic et al. 2003 [49]; Kawaki et al. 2008 [50]
KO Constitutive	CCN2^Δex3−5::LacZ^	Growth plate abnormalities decreased bone density in both appendicular and axial skeletal sites	Lambi et al. 2012 [51]
KO Conditional	CCN2 ^fl/fl^;Tg(Prx1-Cre)	Decreased trabecular bone volume and trabecular number observed at 1 month and resolved by 4 months of age	Canalis et al. 2010 [52]
KO Conditional	CCN2 ^fl/fl^;Tg(Ocn-Cre)	Male-specific osteopenic phenotype observed at 6 months	Canalis et al. 2010 [52]
KI Conditional	Tg(Ocn-CCN2)	Significant decrease in bone mineral density and trabecular bone volume due to impaired osteoblast activity	Smerdel-Ramoya et al. 2008 [53]
CCN3			
KO Constitutive	CCN3^Δex3::TKNeo^	Skeletal overgrowth, enlarged vertebrae, elongated bones, and joint abnormalities	Heath et al. 2008 [54]
KO Constitutive	CCN3^Δ::LacZ−Neo^	Modest skeletal phenotype between 1 and 10 months of age	Canalis et al. 2010 [55]
CCN4			
KO Constitutive	CCN4^EX2::PGK−Neo^	Female Wisp1 KO mice exhibited reduced BMD and trabecular number	Maeda et al. 2015 [20]
KI Constitutive	Tg(Col1a1-CCN4)	Increased BMD, trabecular thickness, and bone volume	Yoshioka et al. 2016 [56]
CCN5			
KO Constitutive	CCN5^Δex2−5::LacZ^	No skeletal phenotype	Jiang et al. 2018 [39]
CCN6			
KO Constitutive	CCN6^Δex3−5::LacZ−neo^	No skeletal phenotype	Kutz et al. 2005 [57]

While global *CCN1* KO is embryonically lethal, severe skeletal deformities caused by germline deletion of *CCN2* result in perinatal mortality by respiratory failure [46, 49]. Constitutive *CCN2* KO demonstrates a profound impact on mouse skeletal morphogenesis, including abnormal organization of the proliferative and hypertrophic zones of the growth plate, which leads to decreased trabecular bone in the metaphysis of appendicular sites and reduced density in caudal vertebral bodies, mandibles, and parietal bones [49, 51]. Analyses of constitutive *CCN3* KO mice have reported contrasting degrees of skeletal abnormalities, possibly resulting from differences in the method of genetic targeting. One model employed a genetic construct which replaced exon 3 of *CCN3* with a TKneomycin cassette, resulting in 129/SvJ-background KO mice (CCN3^Δex3::TKNeo^) that produce no full-length CCN3 protein, instead expressing low-levels of a mutant CCN3 protein lacking the VWC domain [54]. The study reported significant malformations in musculoskeletal development, including embryonic overgrowth of the appendicular and axial skeleton, enlarged vertebrae, elongated long bones and digits, delayed ossification, increased bone mineralization, and severe joint deformities. Analysis at E16.5, E18.5, and E19.5 stages of embryonic development indicated that *CCN3* deletion enhanced both chondrogenesis and osteogenesis. Micromass cultures of embryonic cells demonstrated significantly increased Alcian blue and alkaline phosphatase staining relative to wild-type (WT) controls. Finally, KO mice displayed muscular atrophy by 5 months of age, associated with adipose transdifferentiation. In another study, constitutive *CCN3* KOs were generated from C57BL/6 mice, engineered to replace the entire coding sequence of the CCN3 gene with a LacZ/neomycin (LacZ/Neo) cassette, flanked by LoxP sequences to allow excision of the cassette by Cre recombination (CCN3^Δ::LacZ−Neo^) [55]. KO mice generated by this method were reported to display a modest, sex-specific changes in skeletal phenotype observed between 1 and 10 months of age. Namely, histomorphometric analysis of female KO bones showed an increase in osteoblast surface and mineralizing surface, suggesting a greater bone-forming surface. In contrast, male KO mice exhibited increased osteoclast number and bone resorption at 7 months of age, accompanied by significant increases in serum concentration of C-terminal telopeptide of type I collagen (CTX), a biomarker of bone remodeling, by age 4 months.

The disparity of bone phenotype observed in the two strains of *CCN3* KO mice possibly reflects complexities in CCN signaling mediated by their various fragments, as the former KO mouse continued to express detectable levels of a truncated form of CCN3, while the latter reflected complete deletion of the entire protein coding region. Recent studies have also demonstrated fascinating relationships between the bioactivities of full-length CCNs and their respective fragments [18]. For example, the TSP1 domain of CCN5 was shown to recapitulate the effect of the full-length protein in stimulating estrogen receptor-α expression in MDA-MB- 231 mammary adenocarcinoma cells and inhibiting epithelial-to-mesenchymal transition (EMT) [58]. Interestingly, the CCN5 fragment retained competency in blocking CCN2-stimulated mammosphere formation of MCF- 7 adenocarcinoma cells and scratch wound gap closure in the Rat2 embryonic fibroblast line. In the context of bone signaling, the C-terminal fragment of CCN2 (consisting of TSP1 and CK domains), was shown to reproduce the activity of full-length CCN2 in stimulating RANKL-induced osteoclast differentiation in the RAW264.7 murine macrophage line, while the homo-dimeric form of the fragment exhibited 20-fold higher bioactivity relative to the full-length CCN2 [59]. Based on noted phenotypic distinctions between skeletal KO models, potential differences in the function and bioactivity of full-length CCN3, relative to its fragments, raise intriguing possibilities around complex regulatory mechanisms through which CCNs may modulate bone homeostasis.

Constitutive deletion of *CCN2* and *CCN3* in mice has demonstrated important roles in embryonic skeletal development. In contrast *CCN4* KO mice exhibit significant defects in bone density, trabecular microarchitecture, and mechanical properties, implicating CCN4 as a regulator of bone matrix composition and biomechanical properties, rather than skeletal morphogenesis [19]. Constitutive *CCN4* KO mice were produced by generating a germline frameshift mutation in the coding sequence of the CCN4 protein through insertion of a PGK-neo cassette into an EcoRI site on exon 2 (CCN4^Ex2::PGK−Neo^) [20]. Effectiveness of the targeting strategy was verified in 3-day-old calvarial osteoblasts harvested from KO and WT mice, which showed significant transcriptional downregulation of *CCN4* mRNA by real-time PCR and northern blot, while hindlimb bone protein extracts confirmed absence of CCN4 protein in KO by western blot. Whole-body dual-energy x-ray absorptiometry (DXA) measurements indicated that both male and female KO mice exhibited statistically significantly reductions in BMD, which persisted from 3 to 9 months of age. Micro-computed tomography (μCT) analysis of femoral bone microarchitecture in 3-month-old KO and WT mice demonstrated both sexes of KO mice as exhibiting significantly reduced cortical thickness and cross-sectional area compared to WT, while significant decreases in trabecular bone volume/total volume (BV/TV), trabecular number, and separation were restricted to female KOs relative to WT.

Conditional inactivation of *CCN* genes, driven by osteoblast lineage-associated promoters, has been employed to interrogate the function of CCNs in bone-specific KO mice. This strategy has been particularly important for delineating skeletal mechanisms mediated by CCN1 and CCN2, whose profound impact on embryonic development largely restricts germline-level deletion [46, 49]. In a study comparing phenotypes of transgenic mice of C57Bl/6 J background, in which *CCN1* excision was mediated by various bone-specific promoters driving Cre recombinase, transgenic mice in which Cre activity was controlled by the osteocalcin (OCN) promoter [CCN1^fl/fl^;Tg(Ocn-Cre)], exhibited significantly compromised bone phenotype and perturbations of multiple mechanism regulating bone metabolism [47]. Tissue-specific reduction of *CCN1* mRNA was verified by quantitative real-time PCR from total RNA, isolated from cortical bone samples of 3-month-old KO and WT mice. Comparison of femoral and tibial bones revealed significantly reduced trabecular and cortical mass, diminished vasculature (correlated with reduced VegfA expression), and decreased Wnt signaling accompanied by increased SOST levels, in KO animals relative to WT littermates at 3-months of age. The study also noted increased bone marrow adiposity in KO mice, along with greater osteoclast numbers, accompanied by higher expression of *RANKL* mRNA.

Conditional, bone-specific deletion of *CCN2* was achieved by Cre-mediated excision, in two transgenic mouse models of primarily C57BL/6 background, in which expression of the recombinase was differentially regulated by either the paired-related homeobox 1 (Prx1) enhancer [CCN2^fl/fl^;Tg(Prx1-Cre)] or the OCN promoter [CCN2^fl/fl^;Tg(Ocn-Cre)] [52]. These regulatory elements were selected for genetic targeting of different stages of skeletal development, as the Prx1 enhancer is active in limb buds at embryonic day 10.5 (E10.5), and the osteocalcin promoter is active in osteoblasts at E18.5. Accordingly, bone phenotypes of the KO mice varied depending on the Cre system and the sex of the mice. For both promoters, *CCN2* excision was validated my measuring *CCN2* mRNA from calvarial bone extracts. Femoral histomorphometry of 1-month-old male *CCN2*^*Prx1*^ KO mice demonstrated decreased trabecular bone volume and number, relative to male WT littermates, while development of a similar osteopenic phenotype in male *CCN2*^*OCN*^ KO mice was observed at 6 months of age. In this study, the osteopenic phenotypes attributed to CCN2 deletion were not observed in KO females, suggesting a sexual dimorphism in the impaired endochondral bone formation caused by the conditional deletion of *CCN2* driven by these two promoters. Interestingly, a study characterizing the skeletal impact of CCN2 overexpression in conditional knock-in (KI) mice also controlled by the OCN promoter [Tg(Ocn-CCN2)] similarly reported a degree of sexual dimorphism in the resulting phenotypes [53]. Histomorphometric analysis of distal femurs in 1-month-old transgenic KI mice exhibited decreased trabecular bone volume and osteopenia in both sexes. However, the reduction in bone formation was significantly more pronounced in females. By characterizing sexual dimorphism in skeletal phenotypes arising from OCN-driven excision versus overexpression of CCN2, results from these two studies offer a valuable insight into the complex regulatory balance of bone growth and maintenance in which CCNs appear to serve a critical role. Moreover, the observation that both negative and positive deviations from homeostatic levels of *CCN2* expression produce a deleterious effect on skeletal phenotype further demonstrates the broader theme of CCN proteins as “signal amplifiers” in bone, influencing both anabolic and catabolic processes. Finally, the observed sensitivity of bone mass, structure, and biomechanical properties to alterations in CCNs suggests that perturbations associated with aging or disease may both contribute to the deterioration of bone health and represent prospective therapeutic targets.

## CCNs in Bone Development, Growth, and Repair

CCNs participate in skeletal morphogenesis from embryonic development. Although CCN1 - 5 are all expressed in the murine skeletal anlage, phenotypic results from gene targeted mouse models suggest different levels of participation in bone development [19]. Constitutive KO mice have demonstrated essential roles for CCN1 and CCN2 in embryonic and perinatal survival, whereas CCN2 and CCN3 appear to be necessary for normal bone morphogenesis [46, 49, 51, 54]. Conversely, skeletal phenotypes resulting from global deletion of *CCN4* demonstrate a significant function in regulating bone quality rather than direct participation in bone development [20]. At present, analogous methods of constitutive genetic targeting of *CCN5* and *CCN6* have not produced evidence for their developmental or regulatory functions in bone [39, 57].

Embryonic mortality caused by severe defects in vasculogenesis associated with global *CCN1* KO has largely precluded analysis of the specific roles of CCN1 in skeletal patterning or bone morphogenesis [60]. However, the lethal perinatal phenotype of constitutive *CCN2* KO mice provides important indications regarding its function in regulating cartilage-to-bone transition during endochondral bone formation. Namely, *CCN2* KO mice demonstrated expanded zones of mineralizing cartilage, coupled with reduced ossification of the thoracic skeleton, while histological analyses indicated impaired angiogenesis of the calcified cartilage matrix, suggesting significant inhibition of multiple processes involved in endochondral ossification [49]. Consistent with these findings, studies of cartilage phenotype in constitutive *CCN2* KO mice (CCN2Δ^ex1::PGK−NeopA^) have observed severe chondrodysplasia, resulting from aberrant ECM organization and impaired proliferation and differentiation of chondrocytes [61]. Other reports showed that *CCN2* KO mice exhibited enlarged hypertrophic zones in cartilage growth plates, defective matrix remodeling, impaired vascularization, and diminished osteoid formation by osteoblasts [62]. Together, these findings point to a critical role for CCN2 in coordinating multiple aspects of endochondral ossification, a process fundamental not only to skeletal development but also to bone repair. Moreover, the emerging paradigm that CCN2 modulates both anabolic and catabolic activities may extend to other CCN family members, whose precise functions in bone formation and remodeling remain to be characterized.

Understanding prospective functions of CCNs in bone requires delineation of the two primary processes involved in skeletogenesis: intramembranous and endochondral ossification, which are distinguished by their mechanisms and cellular contributions [63]. Intramembranous ossification is responsible for the morphogenesis of flat bones, such as the skull and clavicle, which are formed by direct osteoblastic differentiation of MSCs without a cartilage intermediary. Analyses of frontal bones and tibiae in global *CCN2* KO mice revealed significantly impaired bone matrix synthesis and reduced osteoblast proliferation and maturation relative to WT littermates, while interparietal bones exhibited a substantially less developed ossified area, suggesting that CCN2 influences osteoblastic functions directing intramembranous ossification [50]. In contrast, endochondral ossification is initially mediated by chondroprogenitors, which lay down a transient cartilage anlage that is substituted for mineralized bone in order to give rise to much of the axial and appendicular skeleton, including vertebrae and long bones. Mineralization of the anlage by hypertrophic chondrocytes, as well as secretion of angiogenic cues such as vascular endothelial growth factor (VEGF), stimulates vascularization of the calcified cartilage matrix [12]. Infiltrating blood vessels convey osteoclast precursors, which initiate resorption of the transitional matrix, while osteoblast precursors are recruited to deposit osteoid in its place. Notably, the reparative phase of bone healing recapitulates many of the key mechanisms active in endochondral ossification, suggesting analogous involvement of CCNs in regulating bone regeneration.

Extensive evidence from transgenic and KO animal models (Table 1), as well as in vitro and cell-based studies, implicates CCNs as critical regulators of lineage commitment in the three principal protagonists of endochondral ossification: chondrocytes, osteoclasts and osteoblasts [19]. Expression patterns of all CCN family members have been identified during various stages of endochondral ossification in mouse postnatal skeletal development [38]. Immunofluorescent staining of WT mouse calvariae harvested at postnatal day 3 revealed localization of CCN1 and CCN2 in osteoblasts and osteocytes near the bone surface, while CCN4 and CCN5 were found in osteocytes located within the bone matrix. Additionally, the study profiled dynamic changes in transcriptional expression of all *CCN* genes over the course of extended, 28-day differentiation cultures of murine primary osteoblasts, observing that CCNs have distinct roles at various stages of osteoblast differentiation. *CCN1* and *CCN2* mRNA transcripts peaked at early stages of ascorbate-induced differentiation. In contrast, *CCN3* expression was highest in undifferentiated osteoblasts and declined steadily following induction, while *CCN4* and *CCN5* mRNA were detected throughout culture period, increasing toward the latter half of differentiation. Low-level transcription of *CCN6* was consistent over the course of culture. Similarly, a study profiled CCN expression during BMP2-induced chondrocyte differentiation of primary human MSC micropellet cultures [64]. While undifferentiated MSCs maintained transcriptional expression of *CCN1 - 5*, chondrocyte induction was associated with significant reductions in *CCN1* and *CCN6* mRNA, while *CCN3* and *CCN4* expression increased two to three-fold. In addition to providing evidence that CCNs may cooperatively direct differentiation of multiple skeletal lineages, these findings suggest that a primary function of CCNs in bone may revolve around integration of signaling cues which coordinate activities of multiple cell types [65]. For example, following epiphyseal plate closure, the primary function of CCNs in bone may shift from coordinating ossification towards balancing the activities of mesenchymal stem MSCs, osteoblasts, and osteoclasts in postnatal bone growth, homeostasis and repair.

## CCNs in the RANKL/RANK/OPG Axis and PTH Signaling

Postnatal skeletal growth and bone remodeling requires coordination of anabolic and catabolic activities through regulatory feedback between MSCs, osteoblasts, osteoclasts, and osteoimmune cells [66]. Receptor activator of nuclear factor κB ligand (RANKL) is a potent stimulator of osteoclastogenesis, belonging to the tumor necrosis factor (TNF) superfamily. Although RANKL is an essential pleiotropic regulator of bone resorption, recent studies indicate that it also influences osteoblastogenesis and MSC differentiation [67]. In primary murine bone marrow MSCs, RANKL-RANK binding suppresses Wnt signaling by accelerating β-catenin degradation and inhibiting its synthesis, resulting in impaired osteoblastic differentiation [68]. In the same study, the bone phenotype of 8-week-old transgenic mice in which conditional KO of RANK in bone marrow MSCs was driven by the *Prx1* promoter, demonstrated increased trabecular bone volume and greater bone mineral density of distal femurs compared to WT littermates. In addition, emerging evidence from both mouse bone marrow stromal cell lines and primary osteoblasts indicates that a reverse form of RANKL signaling, derived from maturing osteoclasts, may alternatively promote osteoblastogenesis by activating the PI3K–Akt–mTORC1 cascade, crucial for osteoblast differentiation via Runx2 [69–71]. Moreover, histomorphometric analyses of lumbar vertebrae from 7-week-old RANKL^P29 A^ mutant mice show significantly reduced bone formation, while osteoclast numbers at the bone surface are unaffected, indicating that suppression of RANKL reverse signaling impairs bone formation under normal physiological conditions [69]. This emergent, bidirectional paradigm of RANKL signaling contributes to the functional coupling of bone resorption and formation and potentially reframes understanding of bone anabolism and catabolism as co-regulated, rather than sequentially distinct, processes [67].

PTH is a calciotropic regulator which also directly modulates both anabolic and catabolic axes of bone metabolism, as intermittent administration promotes osteogenesis by stimulating osteoblastic differentiation of MSCs, while continuous PTH induces bone resorption by enhancing osteoclastogenesis. In its catabolic role, PTH stimulates osteocytes and osteoblasts to secrete RANKL, which promotes maturation of monocyte/macrophage precursors into mature osteoclasts through binding RANK transmembrane receptor [72]. To moderate this catabolic pathway, osteoblasts secrete osteoprotegerin (OPG), a RANKL receptor antagonist which controls osteoclast differentiation by inhibiting binding to RANK. Accordingly, the ratio of RANKL expression relative to OPG is a key mechanism for coupling resorption and formation in balanced bone turnover [73]. While PTH-mediated osteoclastogenesis indirectly enhances bone resorption, PTH can also stimulate bone anabolism, by promoting osteoblastic differentiation of MSCs via multiple mechanisms mediated through binding to parathyroid hormone receptor- 1 (PTH1R) [74]. Under homeostatic conditions, this tightly coordinated system of metabolic feedback and endocrine/paracrine signaling balances bone turnover, maintaining bone mass, density, and structural integrity. CCNs, in turn, may serve as critical signal integrators within these bidirectional systems, coupling cytokines (i.e., RANKL) and hormonal signals (i.e., PTH) in order coordinate anabolic or catabolic “directionality” of cues which govern bone metabolism [75].

Both as cytosolic signals and ECM constituents, CCN proteins participate in PTH and RANK/RANKL/OPG signaling through multiple mechanisms, acting as both downstream transcriptional targets of Wnt/β-catenin signaling and transcriptional modulators of the pathway [48, 76–78]. For example, in mouse fibroblasts and human MSCs, CCN5 is activated by the canonical Wnt/β-catenin pathway and the secreted form of the protein synergistically promotes expression of TCF/LEF. The latter is itself a key transcriptional effector of the canonical pathway, which directs nuclear localization of β-catenin, and thus further enhances transcriptional activity of Wnt-target genes associated with osteoblastic differentiation [79, 80]. Further upstream, CCN1 directs PTH responsiveness of mouse osteoblasts by facilitating integrin-mediated expression of PTH1R and suppressing expression of sclerostin (SOST), a key antagonist of Wnt/β-catenin signaling in bone [48]. CCN1 was also shown to support bone anabolism in mouse cortical osteoblasts/osteocytes by moderating RANKL/OPG [47]. Conversely, studies conducted in the RAW264.7 mouse macrophage line, which differentiates into osteoclasts upon RANKL stimulation, demonstrated that CCN2 amplifies RANK signaling by accelerating RANKL-induced translocation of NF-κB and enhancing activation of p38 mitogen-activated protein kinase (p38 MAPK) and c-Jun N-terminal kinase (JNK) [81]. Moreover, the same study indicated that CCN2 also promotes osteoclastogenesis by directly binding OPG to inhibit its decoy interaction with RANK.

As matricellular proteins, CCNs are secreted by cells and incorporated into the extracellular matrix [82]. In addition, the signaling functions of matrix-bound CCNs may be somewhat distinct from those of their cytosolic forms [83]. Historically, a major obstacle to investigating extracellular signaling functions of CCNs stemmed from the absence of methods for recapitulating tissue-specific matrices. To address this gap, a growing number of studies have employed cell-derived matrices, elaborated *in vitro* by fibril-secreting cells, as an effective method for investigating tissue-specific differences in matrix proteomics and modeling cell–matrix interactions [84–88]. This approach has been particularly useful in probing the architecture, mechanical properties, and molecular cues entailed in the bone stromal niche, and several studies have employed osteoblast-derived matrices to identify and enhance mechanisms involved in MSC differentiation and mineralization [89–92]. Importantly, ECM prepared *in vitro* from human primary osteoblasts demonstrated over 50% homology with the proteome of devitalized human bone, suggesting significant concordance in function and composition for studying the bone niche [93]. Recently, matrices produced from primary human bone stromal cells derived donors of various ages have been used to recapitulate alterations in the bone stromal matrix and resulting changes in cell phenotype associated with geriatric bone [8]. The study showed that matrices produced by cells from younger donors (30–45 years old) significantly enhanced osteogenic differentiation of cultured MSCs, relative to those produced form older donors (60–80 years old). These findings were consistent with observations that cell-derived matrices reproduced certain alterations in extracellular cues associated with physiological aging.

Our group employed a similar approach to determine that human MSCs show reduced responsiveness to osteogenic and adipogenic growth factors (i.e., BMP2 and rosiglitazone), when cultured on matrices produced by primary human bone stromal cells from donors of different ages [21]. Proteomic analyses indicated significant enrichment of CCN1 and CCN2 in ECMs produced by young cells, among other compositional, architectural and mechanical properties. In order to examine prospective mechanisms through which matrix-bound CCN1 regulates cellular responsiveness to growth factors, we described genetic methods to respectively reduce incorporation of CCN1 in the young matrix or increase its enrichment in matrix produced by aging cells. Interestingly, blocking extracellular CCN1 significantly abrogated low-dose BMP2 and IGF1 stimulation of cultured MSCs, while increasing its incorporation in aging ECM restored cellular responsiveness to these growth factors. While the study did not directly investigate the influence of matrix-bound CCN1 on the RANKL/RANK/OPG axis or PTH signaling, stimulation of mesenchymal cells by BMP2 and IGF1 has been shown to modulate RANKL/OPG ratio, while BMP2 synergizes with PTH signaling and promotes expression of PTH receptor, PTH1R. Moreover, a recent study indicated that BMP2 strongly induces expression of CCN1 and CCN2 in MSCs [94]. Taken together, these findings suggest prospective functions of matrix-bound CCNs in regulating growth factor response, which may produce significant downstream implications for RANKL and PTH-mediated pathways critical for maintaining bone health.

## RANKL/RANK/OPG Signaling in Aging Bone

Aging-associated changes in bone structure and function commence at mid-life, preceding the decline in estrogen levels which greatly accelerates female bone loss in menopause [3]. Gradual thinning and increased porosity of aging trabecular and cortical bone is accompanied by infiltration of adipose tissue into bone marrow, both of which contribute to reducing bone strength [95–99]. Adipose remodeling of the marrow niche also alters the regulatory cues within the bone microenvironment, contributing to a progressive uncoupling of the resorption and formation phases of bone turnover, allowing osteoclast-mediated catabolism to predominate. The mechanisms responsible for estrogen-independent skeletal aging have been broadly attributed to inflammaging, or senescence-associated, chronic inflammation which occurs in response to oxidative stress, inflammatory cytokines, or other immunological stimuli within the aging microenvironment [100]. In contrast, skeletal degeneration caused by estrogen-deficiency is mainly conceptualized as acting through the RANKL/RANK/OPG signaling axis due to the concomitant increase in RANKL and reduction in OPG in postmenopausal bone [101, 102]. However, recent research suggests that aging also influences the expression of RANKL/OPG by various bone-resident cells [103–105].

While RANKL is expressed by a host of different skeletal cell types, studies suggest that osteocytes are the primary contributors of RANKL in adult bone, expressing significantly higher levels compared to osteoblasts and bone marrow stromal cells. As such, osteocytes play a major role in regulating bone resorption and remodeling via osteoclastogenesis [106, 107]. Recently, studies comparing osteocyte-enriched cortical bone from young (8-month-old) and old (24-month-old) C57BL/6 mice observed aging-related increased in transcriptional expression of *Tnfsf11* mRNA, which encodes RANKL protein [108]. *Tnfsf11*^*DMP1*^ Conditional KO mice, in which RANKL was genetically disrupted in mature osteocytes, did not exhibit an aging-related increase in expression and maintained higher vertebral and femoral BMD relative to WT littermates. In addition to promoting osteoclastogenesis, increased RANKL in aging bone may also impair MSC osteogenic function [68, 109]. At early stages of osteogenic lineage commitment, MSCs were shown to be responsive to RANKL, as their baseline expression of RANK is positively stimulated by early exposure to the soluble form of the cytokine [109]. In MSCs, RANKL-RANK signaling appears to inhibit osteogenesis by decreasing the expression of osteogenic markers, such as progressive ankylosis protein (ANK) [109]. Increasing RANKL expression in aging MSCs appears to be accompanied by a corresponding decrease in OPG expression [110]. Osteoimmunological studies also indicate that bone marrow-resident B lymphocytes not only maintain intimate regulatory crosstalk with osteoblasts and MSCs but similarly increase the RANKL/OPG expression ratio with aging [103, 111–113].

Even converging, aging-related mechanisms, which promote senescence in geriatric bone, either directly or indirectly influence RANKL/OPG signaling. Recent studies show that senescent murine and human bone MSCs increase RANKL expression through the upregulation of the senescence-associated transcription factor GATA4, validating their findings by overexpressing GATA4 in primary osteoblasts [108]. Senescence of MSCs and osteocytes is also associated with senescence-associated secretory phenotype (SASP) expression, which promotes osteoclast differentiation and stimulates bone resorption [114–116]. This variable secretome profile includes pro-inflammatory cytokines, chemokines, and matrix-remodeling enzymes which perturb multiple mechanisms involved in bone homeostasis: dysregulating osteoimmune functions, promoting MSC adipogenesis, and inhibiting osteoblast differentiation [117–120]. Moreover, aging-associated lipid accumulation in bone marrow increases RANKL expression while suppressing OPG and has been shown to mediate osteoclastogenesis [121, 122].

Outside the aging bone marrow niche, osteocyte senescence and apoptosis, along with changes in sclerostin expression, also contributes to the deterioration of aging bone by creating empty lacunae [123, 124]. Reduced bone loading, commonly associated with disuse in aging, has also been shown to increase expression of RANKL and sclerostin by osteocytes [125–128]. This can exacerbate a degenerative musculoskeletal feedback loop, as stimulation of osteoclast activity liberates Transforming Growth Factor β (TGFβ) from resorbed bone, which further promotes turnover [129]. Disruption of the canalicular network compromises intercellular communication between osteocytes, impairs the mechanosensory function of the bone matrix, and hinders nutrient and waste exchange, exacerbating skeletal fragility and impaired bone remodeling during aging [126, 130, 131]. Interestingly, a recent study employing single-cell RNA sequencing (scRNAseq) to identify subpopulations of osteoblast precursors and predict their differentiation into mature osteoblasts and osteocytes identified that CCN4 is significantly upregulated in osteocyte-like MPC2 cells compared to undifferentiated MSCs, suggesting a role in the transcriptomic profile of mature osteocytes [132]. While it remains to be determined if CCNs are directly involved in mechanisms of osteocyte aging, current evidence suggests that they influence the responsiveness of osteocytes to paracrine signaling stemming from aging-related changes in cytokines such as RANKL, sclerostin, or TGFβ within the bone microenvironment.

## Dysregulation of CCNs in Aging Bone: Signal and Amplitude

**Fig. 2:**
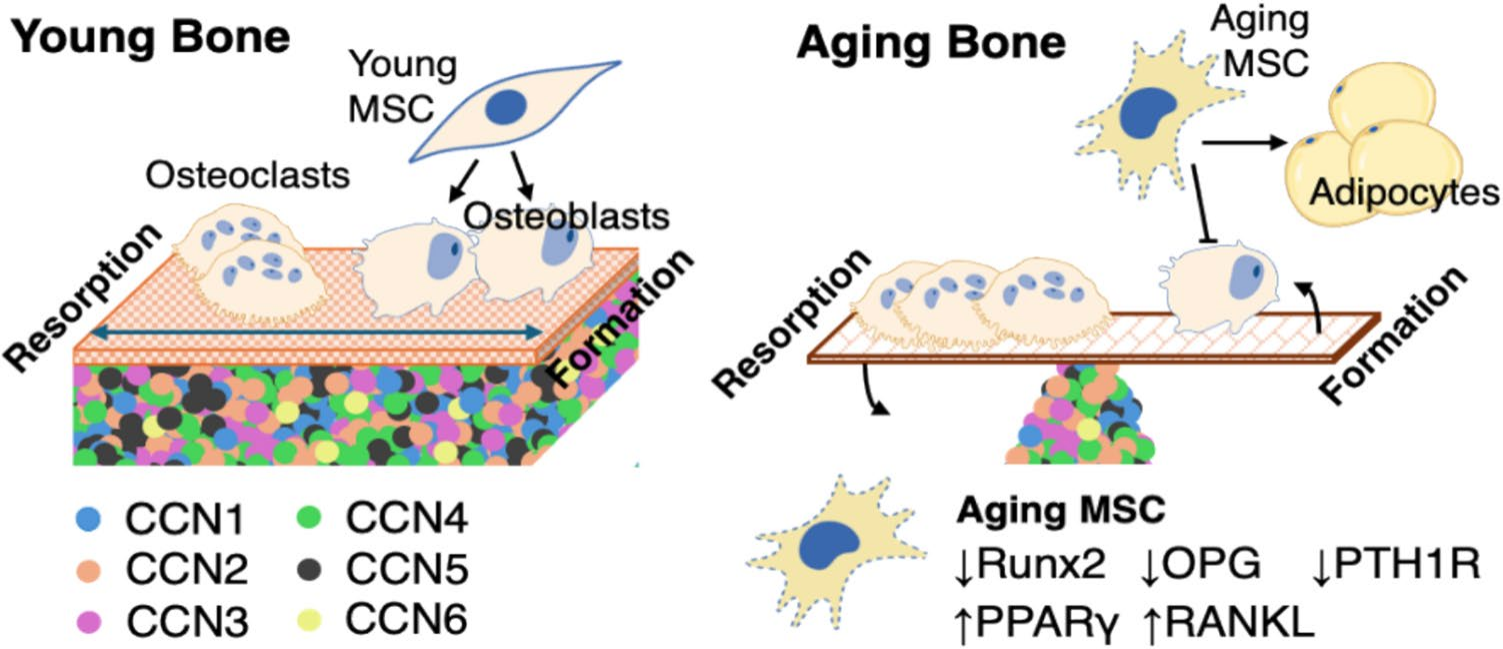
CCN proteins as prospective mediators of degenerative bone aging. Decoupling of bone remodeling during aging produces a delicate metabolic balance, leaving bone highly vulnerable to accelerated degeneration, induced by endocrine changes such as estrogen deficiency postmenopause. This conceptual framework summarizes how aging-associated changes in the accumulation of CCNs may modulate bone homeostasis, dysregulating MSC lineage commitment by modulating responsiveness to PTH signaling and BMPs. CCNs are also implicated in promoting osteoclastogenesis in aging bone via RANKL/OPG. Created using BioRender.com

In contrast to changing expression of cytokines such as RANKL and OPG, which modulate bone metabolism directly through ligand-receptor binding, the contributions of CCNs to aging bone phenotypes may reflect a breakdown in their coordination of cellular responses to those cytokines. In aging, CCNs may amplify or attenuate the effects of various bone regulatory cues, or to extend a proposed conceptual framework for CCN signaling, CCNs tune the “amplitude” of the “signal.” [65] (Fig. 2). Morever, the expression of CCNs themselves may be altered by aging, as shown by recent reports demonstrating significant upregulation of CCN1, along with concomitant decrease in CCN2, in transcriptional and protein analyses of biopsies from aging (> 80 years old) versus young (< 28 years old) human dermis, as well as in primary dermal fibroblasts isolated from the tissue [133, 134]. These studies attributed activation of Mitogen-Activated Protein Kinase (MAPK) pathways and impairment of YAP/TAZ signaling as respectively perturbing expression of CCNs in aging human dermis.

As critical directors of cellular proliferation and apoptosis, dysregulations in p38 MAPK activity, YAP/TAZ nuclear translocation, and Smad signaling cascades have been demonstrated as underlying processes for degenerative phenotypes in aging and osteoporotic bone [135–140]. Significantly, these same pathways are also involved in the regulation of CCN expression [38, 140–143]. Indeed, various reports from skeletal tissues and cells have indicated that aging and degeneration are commonly accompanied by changes in the expression of CCNs [19].

Insights into the role of CCNs in skeletal aging and degenerative disease are currently best exemplified by studies of aging and osteoarthritic cartilage. CCN1 expression in cartilage has been shown to increase in an age-dependent manner, as evidenced by significantly increased immunohistochemical staining in sections of human cartilage from young and aging (> 66 years old) human patients [23]. The same study associated CCN1 expression with histological evidence of aging-related cartilage degeneration, including increased CCN1 accumulation within the superficial matrix and CCN1-positive chondrocytes. These observations were consistent with analyses of tissues and primary chondrocytes isolated from osteoarthritic donors, concluding that CCN1 is a significant driver of aging processes in cartilage, promoting senescence and inflammation, via production of reactive oxygen species (ROS), activation of the p38 MAPK pathway, and increased expression of SASP components, including matrix metalloproteinase 13 (MMP13). Another study identified CCN3 as a biomarker of chondrocyte senescence, reporting aging-associated upregulation in transcriptional and protein expression, as well as increased immunohistochemical staining, in human articular cartilage samples ranging from 19 to 91 years-old [22]. Similarly to CCN1, overexpression of CCN3 in aging cartilage may also be associated with inflammation and osteoarthritic degeneration [144].

While prospective mechanisms of CCNs in aging-related bone loss have not been as extensively characterized, evidence supporting their relationship with aging and osteoporosis is beginning to emerge. For example, transcriptional profiling of primary MSCs, isolated from healthy rat femurs of various ages, compared to those from osteoporotic rats, indicated significant changes in CCN1 expression associated with age and observed the lowest expression of CCN1 in cells harvested from osteoporotic femurs [145]. These results are somewhat consistent with our observation that human primary bone MSCs demonstrate aging-associated changes in the incorporation of CCNs during *in vitro* matrix synthesis [21]. Recent studies provide intriguing insights into the role of CCN1 in aging bone through an investigation of the therapeutic potential of sildenafil for stimulating bone regeneration in an atrophic non-union model performed in 12–15-week-old mice. The results indicated that improved healing of femoral segmental defects in sildenafil-treated mice was accompanied by significant increases in the expression of CCN1, identified in protein extracts harvested from callus tissue. Sildenafil treatment was shown to delay resorption at an early stage of bone regeneration by decreasing osteoclast activity, as indicated by significantly decreased RANKL/OPG ratio at 2 weeks post-surgery, while promoting osteogenic and angiogenic signaling by CCN1 and VEGF. However, a follow-up investigation of aging bone healing conducted by a femoral fracture model in older mice (16–18 months old), showed sildenafil treatment was not effective for promoting bone healing relative to saline controls [146]. The significant reduction in treatment effectiveness in aging mice was attributed to the absence of increased CCN1 in the callus tissue in response to sildenafil-treatment; suggesting dysregulation of mechanisms promoting CCN1 in aging bone. Finally, a recent report used datasets from genome-wide association studies (GWAS) of over 300,000 and 200,000 osteoporosis patients in the United Kingdom and Finland (respectively), to predict serum levels of IGF system members using Mendelian randomization to infer causality [24]. The results of the study predicted that higher serum levels of CCN1 are associated with an increased risk of osteoporosis in both UK Biobank and FinnGen datasets, while the former also projected a negative association between serum CCN2 and osteoporosis risk. Although a great deal remains to be investigated regarding the role of CCN proteins in the pathophysiology of skeletal aging and disease, recent findings suggest broad potential as therapeutic candidates, diagnostic biomarkers, and precision orthobiologics.

## Future Perspectives

Despite their recognized roles in skeletal development and homeostasis, the influence of CCNs on skeletal aging remains largely unexplored. However, emerging evidence suggests that alterations in the accumulation or bioactivity of CCN proteins in bone may contribute to the decoupling of homeostatic mechanisms which coordinate the activates of MSCs, osteoblasts, osteoclasts, and osteoimmune actors [19]. In addition, dysregulation of bone matricellular cues such as CCNs may accelerate bone loss and exacerbate deleterious skeletal effects of declining sex hormone levels by impairing balanced bone turnover.

A major challenge in studying matricellular signaling by CCNs and their role in tissue physiology involves distinguishing between intracellular versus extracellular mechanisms, as well as accounting for potential distinctions in the activity of their discrete domains versus multiple, biologically active fragments [18]. This distinction is particularly significant in bone physiology, because temporal changes in cytosolic expression of CCNs may not directly correspond to accumulation in the surrounding matrix. For this reason, in vitro surrogates, which mimic the bone marrow extracellular niche, are essential for investigating the nuanced roles of CCNs in aging bone [21].

Recapitulation of the extracellular matrix *in vitro* offers a potential strategy for studying the role of CCN proteins within representative matricellular contexts. This approach has been used to reproduce both the bone and adipose-specific progenitor niches *ex vivo* and for studying age-related changes bone matrix proteome, as well as their impact on the function of bone MSCs *in vitro* [21, 91, 92]. Moreover, loss of CCN1 from the extracellular matrix can be reversed, restoring the osteogenic potential of aging bone marrow MSCs; modulation of CCN proteins may represent a new treatment paradigm for restoring the function of aging cells and tissues [114, 147].

Prospective strategies for harnessing CCNs in skeletal health and orthopedic medicine may involve systemic delivery of peptides, monoclonal antibodies, or gene therapies to modulate their expression. Another potential approach includes incorporating of CCN-derived peptides or functional domains into implanted scaffolds as orthobiologic adjuncts to enhance bone healing. For example, a recent investigation demonstrated the osteoanabolic potential of CCN3 to significantly increase bone mass in both ovariectomized and aged female mice [148]. The study employed systemic administration of adeno-associated virus vectors to enhance circulating CCN3 by inducing ectopic expression in hepatocytes. Another recent report demonstrated that the anti-fibrotic potential of the CCN5 TSP1 domain to reverse trans-differentiation of myofibroblasts and reduce the expression of fibrosis markers such as α-SMA and fibronectin [31]. Finally, studies have identified CCN1 as a primary target of different small molecules which were shown to variously promote bone formation, accelerate fracture healing, and enhance bone mineral density and microarchitecture [149–151]. In the immediate future, new modalities in machine learning and computational analyses of multidomain CCN structures and their fragments may be leveraged to map the complex interactome networks of CCN proteins and potentially identify novel binding partners with therapeutic potential [102]. As insights into the multifaceted roles of CCN proteins in skeletal biology continue to emerge, their potential as therapeutic targets for attenuating bone loss or precision orthobiologics for stimulating endogenous skeletal healing will continue to come into focus.

## Key References

- Monsen VT, Attramadal H. Structural insights into regulation of CCN protein activities and functions. J Cell Commun Signal. 2023;17:371–90.
  - ⚬ An excellent overview of emerging evidence indicating that CCN proteins are synthesized as inactive preproproteins requiring proteolytic activation. The review highlights the bioactivity of CCN fragments compared to full-length proteins.
- Marinkovic M, Dai Q, Gonzalez AO, Tran ON, Block TJ, Harris SE, et al. Matrix-bound Cyr61/CCN1 is required to retain the properties of the bone marrow mesenchymal stem cell niche but is depleted with aging. Matrix Biol. 2022;111:108–32.
  - ⚬ The study demonstrates that extracellular matrix-bound CCN1 influences the responsiveness of MSCs to key osteogenic growth factors and highlights its role in aging-related changes within the bone marrow niche.
- Giusti V, Scotlandi K. CCN proteins in the musculoskeletal system: current understanding and challenges in physiology and pathology. J Cell Commun Signal. 2021;15:545–66
  - ⚬ A detailed summary of current knowledge regarding CCN proteins as mediators of musculoskeletal inflammatory pathologies and their role in shaping the tumor microenvironment, with particular emphasis on findings from transgenic models.
- Zhao G, Kim EW, Jiang J, Bhoot C, Charles KR, Baek J, et al. CCN1/Cyr61 is required in osteoblasts for responsiveness to the anabolic activity of PTH. J Bone Miner Res. 2020;35:2289–300.
  - ⚬ This study establishes a key role for CCN1 in bone anabolism through regulation of PTH signaling. CCN1 modulates the expression and activity of PTH1R via interactions with specific integrins.
- Ambrosi TH, Marecic O, McArdle A, Sinha R, Gulati GS, Tong X, et al. Aged skeletal stem cells generate an inflammatory degenerative niche. Nature. 2021;597:256–62.
  - ⚬ An important demonstration that aging-related changes in the secretome and extracellular matrix of skeletal stem cells synergize with RANKL signaling to promote osteoclastogenesis and inflammaging of the aging skeletal niche.
- Menger MM, Emmerich M, Scheuer C, Hans S, Braun BJ, Herath SC, et al. Sildenafil delays bone remodeling of fractured femora in aged mice by reducing the number and activity of osteoclasts within the callus tissue. Biomed Pharmacother. 2024;173:116291.
  - ⚬ This interesting study suggests that, unlike in young mice, where a vasodilator accelerates fracture healing by promoting CCN1, treatment does not up-regulate CCN1 in older mice and similarly fails to improve bone healing.

## Data Availability

No datasets were generated or analysed during the current study.
